# A Prospective Randomized Controlled Trial Investigating Quadriceps Versus Hamstring Tendon Autograft in Anterior Cruciate Ligament Reconstruction

**DOI:** 10.1177/03635465231222279

**Published:** 2024-01-29

**Authors:** Jay R. Ebert, Nicholas D. Calvert, Ross Radic

**Affiliations:** † School of Human Sciences (Exercise and Sport Science), University of Western Australia, Perth, Western Australia, Australia; ‡ HFRC Rehabilitation Clinic, Perth, Western Australia, Australia; § Perth Orthopaedic & Sports Medicine Research Institute, Perth, Western Australia, Australia; ‖ Department of Orthopaedics, Royal Perth Hospital, Perth, Western Australia, Australia; ¶ Perth Orthopaedic & Sports Medicine Centre, Perth, Western Australia, Australia; # School of Medicine, University of Western Australia, Perth, Western Australia, Australia; Investigation performed at the School of Human Sciences (Exercise and Sport Science), University of Western Australia, Perth, Western Australia, Australia

**Keywords:** anterior cruciate ligament reconstruction, clinical outcomes, hamstring autograft, knee function, quadriceps autograft, retear, return to sport

## Abstract

**Background:**

Numerous graft options are available when undertaking anterior cruciate ligament (ACL) reconstruction (ACLR), although a lack of high-quality evidence exists comparing quadriceps (QT) and hamstring (HT) autografts.

**Purpose:**

To investigate patient outcomes in patients undergoing HT versus QT ACLR.

**Study Design:**

Randomized controlled trial; Level of evidence, 1.

**Methods:**

After recruitment and randomization, 112 patients (HT = 55; QT = 57) underwent ACLR. Patients were assessed pre- and postoperatively (6 weeks and 3, 6, 12, and 24 months), with a range of patient-reported outcome measures (PROMs), graft laxity (KT-1000 arthrometer; primary outcome variable), active knee flexion and extension range of motion (ROM), peak isokinetic knee extensor and flexor strength, and a 6-hop performance battery. Limb symmetry indices (LSIs) were calculated for strength and hop measures. Secondary procedures, ACL retears, and contralateral ACL tears were reported.

**Results:**

All PROMs and knee ROM measures significantly improved (*P* < .0001), and no other group differences (*P* > .05) were observed—apart from the Anterior Cruciate Ligament Return to Sport after Injury (ACL-RSI) score, which was significantly better in the HT group at 3 (*P* = .008), 6 (*P* = .010), and 12 (*P* = .014) months. No significant changes were observed in side-to-side laxity from 6 to 24 months (*P* = .105), and no group differences were observed (*P* = .487) at 6 (HT mean, 1.2; QT mean, 1.3), 12 (HT mean, 1.1; QT mean, 1.3), and 24 (HT mean, 1.1; QT mean, 1.2) months. While the HT group demonstrated significantly greater (*P* < .05) quadriceps strength LSIs at 6 and 12 months, the QT group showed significantly greater (*P* < .05) hamstring strength LSIs at 6, 12, and 24 months. The HT group showed significantly greater (*P* < .05) LSIs for the single horizontal (6 months), lateral (6 and 12 months), and medial (6 months) hop tests for distance. Up until 24 months, 1 patient (QT at 22 months) had a retear, with 2 contralateral ACL tears (QT at 19 months; HT at 23 months). Secondary procedures included 5 in the HT group (manipulation under anesthesia, notch debridement, meniscal repair, and knee arthroscopy for scar tissue) and 6 in the QT group (notch debridement, meniscal repair, knee arthroscopy for scar tissue, tibial tubercle transfer, and osteochondral autologous transplantation).

**Conclusion:**

Apart from the ACL-RSI, the 2 autograft groups compared well for PROMs, knee ROM, and laxity. However, greater hamstring strength LSIs were observed for the QT cohort, with greater quadriceps strength (and hop test) LSIs in the HT cohort. The longer-term review will continue to evaluate return to sports and later-stage reinjury between the 2 graft constructs.

**Registration:**

ACTRN12618001520224p (Australian New Zealand Clinical Trials Registry).

Anterior cruciate ligament (ACL) reconstruction (ACLR) remains the standard treatment in patients experiencing ACL injury.^48^ After surgery, in those who return to sports (RTS), an ipsilateral reinjury rate of 7% has been reported, along with an 8% incidence of contralateral ACL tear.^58^ The reasons for reinjury are multifactorial^45^ and may include graft failure due to inadequate graft selection and/or incorporation, surgical error, inadequate rehabilitation, and/or suboptimal recovery of lower limb strength and functional capacity. ACLR employing a hamstring tendon (HT) or bone–patellar tendon–bone (BPTB) graft has traditionally been a more common option,^35^ with systematic reviews and meta-analyses generally reporting comparable clinical and functional outcomes,^6,41,46^ inclusive of knee stability, graft failure, and patient-reported outcome measures (PROMs). However, some studies have reported greater knee laxity and/or reinjury rate after HT ACLR,^7,32,46^ albeit a lower risk of anterior knee pain and kneeling discomfort has been reported in those undergoing HT ACLR.^6,32,41^

Given the aforementioned findings, particularly with respect to graft harvest site burden, HT ACLR has been a popular choice, although an increasing interest has developed in using a quadriceps tendon (QT) autograft.^53^ A recent biomechanical cadaveric study^16^ reported a comparative ultimate load to failure between HT, QT, and BPTB grafts, with favorable structural properties in QT grafts compared with other grafts and greater stiffness compared with HT grafts. Even though comparative data remain in relative infancy, systematic reviews and meta-analyses published thus far comparing QT ACLR with other graft constructs have generally reported no differences in PROMs, knee stability, graft failure, and/or revision rates.^1,5,20,36,37,43,50,51^ However, studies have also reported less graft harvest site pain and/or donor-related symptoms in QT versus BPTB^1,36,43,50^ and HT^21^ ACLR, better functional outcomes in QT versus HT ACLR,^36^ less knee laxity in QT versus HT ACLR,^5,40^ and a lower rerupture rate in QT versus HT ACLR.^21^ Although a thorough assessment of the recovery of strength and function is yet to be reported, Ajrawat et al^1^ reported an ongoing need for well-designed randomized controlled clinical trials (RCTs) comparing QT with other autografts.

Previous research has focused on comparing outcomes after HT and BPTB ACLR—although there remains a current lack of high-quality evidence comparing HT and QT ACLR outcomes. This study aimed to conduct a robust subjective and objective assessment of patients undergoing ACLR, randomized to an HT or QT autograft. It was hypothesized that (1) no differences between groups would exist in graft laxity, (2) the incidence of graft failure and contralateral ACL rupture would be similar across the 2 groups, (3) deficits in peak isokinetic knee extensor and flexor strength—as reported via limb symmetry indices (LSIs)—would be relevant to the graft harvest site (ie, knee extensor deficits in QT ACLR; knee flexor deficits in HT ACLR) at 6 months after surgery—although they would have resolved by 12 months, (4) no group differences would be observed for hop test LSIs, and (5) no differences between groups would exist in commonly employed PROMs.

## Methods

### Participants and Recruitment

Patients scheduled to undergo ACLR via a single surgeon (R.R.), with or without concomitant meniscal pathology, were recruited for the study. This parallel group study randomized patients to either an HT (n = 57) or QT (n = 59) autograft construct using a “random number sequence generator.” The random number sequence was maintained by an independent researcher, with each group randomization released upon recruitment and consent. Patients were included if they were aged 16 to 50 years and qualified for ACLR based on clinical examination and magnetic resonance imaging. The exclusion criteria were as follows: unable or unwilling to sign the patient informed consent specific to this study; a body mass index of ≥40; revision or multiligamentous reconstruction; presenting with significant articular cartilage pathology that likely required concomitant surgical intervention; and being treated for a psychiatric disorder, senile dementia, Alzheimer disease, or the presence of alcohol or substance abuse. Ethical approval was provided by the Hollywood Private Hospital (HPH541) Human Research Ethics Committee, and the trial was conducted according to the Declaration of Helsinki. The trial was registered in the Australian New Zealand Clinical Trials Registry (ANZCTR - ACTRN126180015 20224p).

### ACLR Surgical Techniques

All ACLR procedures were performed by a single surgeon with patients under general anesthesia. All patients had a standardized anesthetic regimen, consisting of a single-shot adductor canal regional block using 0.375% ropivacaine plus 8 mg dexamethasone, placed using ultrasound guidance once the patient was under general anesthetic.

For the HT group, graft harvest was via a transverse incision over the pes anserinus using a closed tendon harvester, harvesting the semitendinosus that was quadrupled and prepared. The Arthrex Graftlink system (Arthrex) was employed, enabling a single HT harvest for over 90% of cases. In a minority of cases, the gracilis was harvested and used in combination to create a minimum graft diameter of 8 mm in all patients. In the QT group, an all–soft tissue graft was harvested via a longitudinal incision over the distal portion of the quadriceps tendon and proximal pole of the patella. The soft tissue graft was obtained using a 9-, 10-, or 11-mm graft harvester depending on the patient's size. Approximately 70 mm of graft was harvested and prepared again as per the Arthrex Graftlink system guidelines.

Patients underwent knee ligamentous examination under anesthesia, followed by a diagnostic arthroscopy to evaluate meniscal and/or chondral damage, and surgery was performed accordingly if required. Any concomitant surgery (eg, meniscectomy or meniscal repair; simple chondroplasty, if required) was performed before ACLR. Femoral and tibial tunnels were prepared to allow for passage of the graft in an anterograde manner using adjustable loop fixation for both the femoral and tibial fixation. Femoral tunnel preparation was made utilizing an inside-out technique, drilling via the anteromedial portal with a line-to-line fit. Graft tensioning was performed in full knee extension in line with the manufacturer's recommendations, with the cycling of the graft before final fixation. The wounds were closed in layers, hemostasis was ensured, and absorbable subcuticular wound closure was employed. Dressings were applied, followed by wool and crepe bandages. Standardized analgesic regimens postoperatively consisted of regular paracetamol, non-steroidal anti-inflammatory medications, and stronger analgesics for breakthrough pain, while standardized education was provided to all patients regarding regular icing, compression, and limb elevation for swelling control.

### Rehabilitation

Early (first 2 weeks) rehabilitation advice was focused on pain/swelling reduction, safe and appropriate crutch ambulation, and exercises to improve patellar mobility, early knee range of motion (ROM), and quadriceps control. Generally, the weightbearing status of patients who underwent concomitant meniscal repair was modified, with a gradual reduction in the use of crutches and bracing over the first 6 weeks. More specifically, unstable meniscal tears (radial tears) were treated with up to 6 weeks of restricted weightbearing, whereas patients with stable meniscal tears were allowed to bear weight immediately after the repair as tolerated. Early hydrotherapy was advocated after the 2-week orthopaedic review for all patients and pending the healing of surgical incisions. Although rehabilitation in the initial 6 weeks was relatively conservative to permit pain and swelling reduction, progressive strengthening exercises were initiated 6 weeks after surgery. For this study, rehabilitation was not standardized for 6 weeks and was undertaken under the direction of the patient's own outpatient physical therapist. Furthermore, there was no later-stage standardization with respect to the initiation of higher-level loading, plyometric, agility, and/or sport-specific training activities, and there were no standardized criteria that permitted RTS.

### Clinical Assessment

Several PROMs were assessed presurgery and at 3, 6, 12, and 24 months after surgery—including the International Knee Documentation Committee Subjective Knee Evaluation Form,^23^ the Knee Outcome Survey Activities of Daily Living Scale,^24^ the Lysholm scale,^33^ the Cincinnati Knee Rating System,^4^ and the Tegner Activity Scale.^52^ Furthermore, other PROMs were completed at various postoperative time points, including a pain visual analog scale (VAS) for the knee and pain VAS for graft harvest site with scores from 0 (no pain) to 10 (severe pain); the Anterior Cruciate Ligament Return to Sport after Injury (ACL-RSI)^55^ score; the donor site–related functional problems after anterior cruciate ligament reconstruction score^3^; and a Global Rating of Change scale,^28^ evaluating the patient's perceived status compared with before the injury, ranging from −5 (very much worse) to 0 (about the same) to 5 (completely recovered).

Anterior tibial translation (in millimeters) during a maximal manual test was evaluated on both limbs via the KT-1000 knee arthrometer (MEDmetric Corp)^42^ at 6, 12, and 24 months, while active knee flexion and extension ROM (in degrees) was assessed on the operated limb at 6 weeks and 3, 6, 12, and 24 months using a long-arm goniometer. At 6, 12, and 24 months after surgery, patients underwent a 6-hop test battery that included (1) the single horizontal hop for distance (SHD; in meters), (2) the 6-m timed hop (in seconds), (3) the triple hop for distance (in meters), (4) the triple crossover hop for distance (in meters), (5) the single lateral hop for distance (LHD; in meters), and (6) the single medial hop for distance (MHD; in meters). Peak isokinetic knee extensor and flexor strength (in Newton-meters) were measured at 90 deg/s using an isokinetic dynamometer (Isosport International).

All clinical reviews were conducted within the same private outpatient physical therapy clinic. Although patients could not be blinded to their group allocation, the varied location of scars between the 2 groups made it difficult to blind the physical therapist undertaking the assessments.

### Data and Statistical Analysis

For this prospective RCT, a priori power calculation was determined based on the primary outcome variable (anterior translation via the KT-1000 arthrometer) between the 2 surgical groups. The study was powered to detect a minimum 1 mm difference in laxity, adopted from a previously published study.^15^ Therefore, 102 patients (51 in each group) were required to reveal differences at the 5% significance level, with a power of 80%.

Intention-to-treat was the primary method for data analysis. For the hop and strength measures, LSIs were calculated as a measure of the operated limb as a percentage of the nonoperated limb. The mean (SD) of all pre- and postoperative PROMs were calculated and presented, as were the means (SDs) for laxity, knee flexion and extension ROM, and hop and strength LSIs. Repeated-measures analysis of variance was employed to assess the differences between the 2 groups over time in all measures. Where a significant group or interaction effect was found, the post hoc independent *t* test was used to determine time points at which the 2 groups differed. KT-1000 arthrometer side-to-side difference (in millimeters) scores were further categorized based on the side-to-side difference as normal (<3 mm), nearly normal (3-5 mm), abnormal (6-10 mm), and severely abnormal (>10 mm).^38^ Between-group differences in the percentage of patients participating in their preinjury pivoting sports at 24 months were assessed using the chi-square test. Ipsilateral retears, contralateral ACL tears, and secondary surgical procedures were reported. Where relevant, statistical analysis was performed using SPSS software Version 27.0 (SPSS Inc), with significance determined at *P* < .05.

## Results

**Figure 1.**
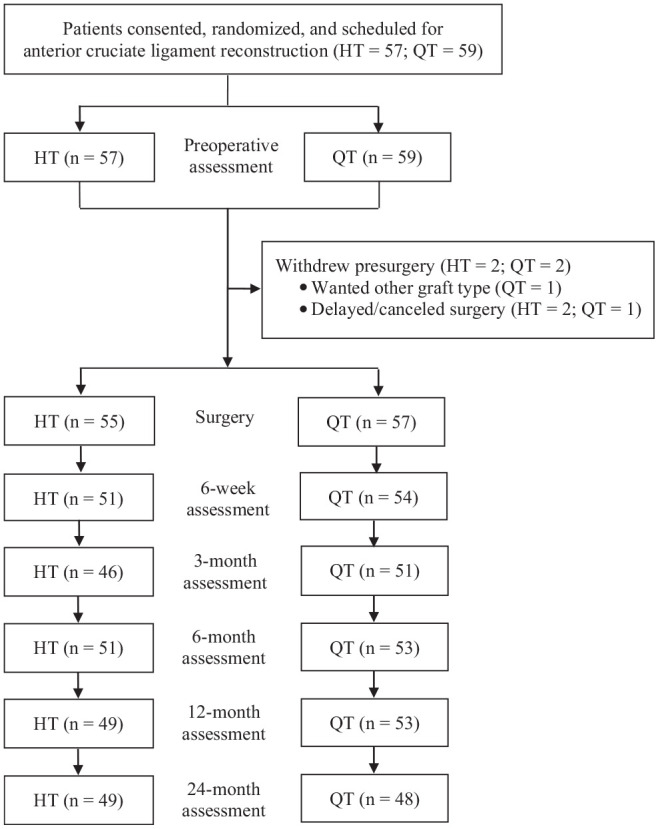
Flowchart demonstrating patient recruitment and evaluation over the 24-month postoperative period in patients randomized to the HT and QT groups. HT, hamstring; QT, quadriceps.

Between March 2019 and December 2020, a total of 116 patients were recruited and randomized for the study (Table 1), of whom 112 patients proceeded to surgery (Figure 1). Of the 112 patients (HT = 55; QT = 57) who were recruited and underwent surgery, 97 patients (HT = 49; QT = 48) were assessed specifically at the 24-month review. For the following reasons, cases were not able to be evaluated during the final 24-month review: ipsilateral retear (n = 1: HT = 0; QT = 1); contralateral ACL tear (n = 2: HT = 1; QT = 1); a secondary orthopaedic knee procedure that did not involve the ACL directly as outlined below, although it did prevent adequate review (n = 2: HT = 0; QT = 2); or inability to attend because of conflicting health issues, geographical relocation, or inability to be contacted (n = 10: HT = 5; QT = 5).

**Table 1:** Characteristics of Patients Randomized to the HT and QT Tendon Autograft Groups Who Were Recruited, Randomized, and Underwent Surgery^*a*^

Variable	Measure	HT (n = 55)	QT (n = 57)
Age, years	Mean (SD)	29.4 (7.7)	28.1 (8.2)
	Range	16-43	16-47
BMI	Mean (SD)	26.3 (3.6)	26.6 (3.6)
	Range	21.4-35.2	19-36.9
Injury to surgery time, weeks	Mean (SD)	9.6 (8.7)	8.5 (8.8)
	Range	1-36	2-48
Sex, male	n (%)	28 (50.9)	28 (49.1)
Injury mechanism, noncontact	n (%)	44 (80)	46 (80.7)
Concomitant surgery	n (%)	34 (61.8)	32 (52.6)
Meniscectomy	n (%)	2 (3.6)	00 (0)
Meniscal repair	n (%)	32 (58.2)	32 (52.6)

a BMI, body mass index; HT, hamstring; QT, quadriceps.

### Subjective Outcomes

**Figure 2. fig2-03635465231222279:**
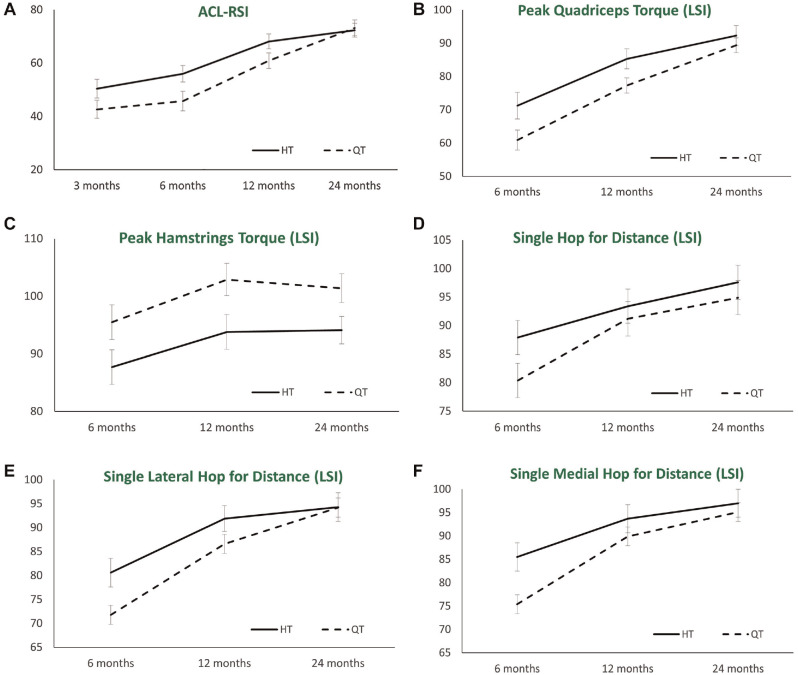
Graphical representation of the HT and QT tendon autograft groups throughout the postoperative timeline for the (A) ACL-RSI, (B) quadriceps strength LSI, (C) hamstring strength LSI, (D) single hop for distance LSI, (E) single lateral hop for distance LSI, and (F) single medial hop for distance LSI. ACL-RSI, Anterior Cruciate Ligament Return to Sport after Injury score; HT, hamstring; LSI, limb symmetry index; QT, quadriceps.

All PROMs significantly improved (*P* < .05) over the 24-month period. The only difference between groups was a significant group effect for the ACL-RSI (*P* = .040) (Table 2). Post hoc *t* tests demonstrated significantly better (*P* < .05) ACL-RSI scores in the HT group at 3 (*P* = .008), 6 (*P* = .010), and 12 (*P* = .014) months after surgery (Figure 2A).

**Table 2:** PROMs Throughout the Pre- and Postoperative Timeline for the HT and QT Tendon Autograft Groups^*a*^

Time Point	Group	IKDC	Lysholm	Cincinnati	KOS	Tegner	VAS–Knee	VAS–Graft	ACL-RSI	Donor-site Score	GRC
Presurgery	HT	50.6 (18.9)	63.8 (21.3)	53.1 (21)	60.1 (19.9)	3.2 (1.5)	N/A	N/A	N/A	N/A	N/A
	QT	49.7 (17.9)	58.9 (23.2)	49.5 (22.5)	56.3 (17.4)	3.2 (1.7)					
6 weeks	HT	N/A	N/A	N/A	N/A	N/A	2.7 (1.9)	1.7 (1.7)	N/A	N/A	N/A
	QT						2.4 (1.8)	2 (1.8)			
3 months	HT	62 (13.3)	75.7 (15.2)	64.2 (13.3)	71.1 (11.3)	3.6 (1.2)	2.3 (1.8)	1.3 (1.3)	50.4 (25.6)	N/A	N/A
	QT	60 (12.3)	75 (13.6)	63.9 (12.7)	69.7 (10.8)	3.5 (1.1)	1.8 (1.4)	1.5 (1.6)	42.6 (22.3)		
6 months	HT	74.5 (12.5)	85 (11.5)	80.4 (12.2)	72.5 (5.9)	4.7 (1.2)	1.4 (1.6)	0.8 (1)	56 (25.2)	22.4 (11.6)	2.2 (1.7)
	QT	71.6 (14.8)	81.2 (12)	75.6 (13.7)	69 (8.6)	4.8 (1.7)	2 (1.7)	1.3 (1.4)	45.7 (25.2)	24.9 (15.3)	1.9 (2.2)
12 months	HT	86.1 (10.8)	89.9 (9.1)	88.9 (11)	76.4 (5.6)	6.3 (1.8)	1 (1.1)	0.9 (1)	68.1 (25.8)	17.2 (12.2)	3.6 (1.2)
	QT	82 (14.2)	88.3 (11.1)	86.1 (12.9)	73.8 (6.8)	6 (1.7)	1.3 (1.5)	1 (1.3)	60.9 (24.7)	15.2 (13.3)	3.1 (1.7)
24 months	HT	91.7 (7.5)	94.4 (6.6)	94.3 (7.2)	77.5 (3.2)	6.8 (1.7)	0.7 (0.8)	0.7 (0.9)	73.4 (26.5)	12.3 (10.7)	4.1 (0.9)
	QT	89.1 (9.6)	92.5 (8.0)	92.8 (8.7)	76.5 (3.6)	6.7 (1.4)	0.9 (1.2)	0.8 (1.1)	72.4 (21.9)	9.3 (10.6)	3.8 (1.4)
Time effect, *P*		**<.0001**	**<.0001**	**<.0001**	**<.0001**	**<.0001**	**<.0001**	**<.0001**	**<.0001**	**<.0001**	**<.0001**
Group effect, *P*		.447	.206	.391	.124	.679	.776	.875	**.040**	.301	.125
Interaction effect, *P*		.715	.208	.473	.349	.998	.861	.456	.193	.316	.797

a Data are presented as mean (SD). Bold values indicate statistically significant *P* values. ACL-RSI, Anterior Cruciate Ligament Return to Sport after Injury Score; Cincinnati, Cincinnati Knee Rating System; GRC, Global Rating of Change; HT, hamstring; IKDC, International Knee Documentation Committee Subjective Knee Evaluation Form; KOS, Knee Outcome Survey; N/A, not applicable; Pre, preoperative; PROM, patient-reported outcome measure; QT, quadriceps; VAS, pain visual analog scale.

### Objective Outcomes

No group differences were observed in side-to-side laxity, and there was no significant change in side-to-side laxity from 6 to 24 months (Table 3). At 24 months, KT-1000 knee arthrometer scores demonstrated normal (<3 mm) side-to-side differences in 94% and 92% of the HT and QT groups, respectively (Table 4). Knee flexion and extension ROM significantly improved, with no group differences observed (Table 3). All isokinetic strength and hop test LSIs significantly improved (*P* < .05) (Table 5). A significant group effect was observed that favored greater quadriceps strength LSIs in the HT group (Table 5), with post hoc *t* tests demonstrating a significantly greater (*P* < .05) quadriceps strength LSI in the HT group at 6 and 12 months (Figure 2B). A significant group effect was observed that favored greater hamstring strength LSIs in the QT group (Table 5), with post hoc *t* tests demonstrating a significantly greater (*P* < .05) hamstring strength LSI in the QT group at 6, 12, and 24 months (Figure 2C). After this, a significant group effect was observed for the SHD LSI (*P* = .026), LHD LSI (*P* = .046), and MHD LSI (*P* = .007) (Table 5), with post hoc *t* test demonstrating significantly greater (*P* < .05) LSIs for the SHD (6 months), LHD (6 and 12 months), and MHD (6 months) in the HT group (Figure 2, D [Fig fig2-03635465231222279]-F).

**Table 3:** KT-1000 Knee Arthrometer Side-to-Side Difference Scores and Knee ROM (Flexion and Extension) Outcomes for the HT and QT Tendon Autograft Groups Throughout the Postoperative Timeline^*a*^

Time Point	Group	Knee Flexion, deg	Knee Extension, deg	Laxity, Side-to-Side Difference, mm
6 weeks	HT	107.2 (21.2)	2.1 (3.6)	N/A
	QT	108.0 (21.6)	0.8 (2.5)	
3 months	HT	119.1 (36.1)	0.2 (2.9)	N/A
	QT	126.8 (15.2)	0.5 (2.1)	
6 months	HT	140.6 (8.1)	−0.1 (3)	1.2 (0.9)
	QT	141.9 (6.6)	−0.3 (1.8)	1.3 (1.4)
12 months	HT	144.6 (6.7)	−0.7 (2.3)	1.1 (0.9)
	QT	146.1 (4.8)	−0.8 (1.7)	1.3 (1.2)
24 months	HT	147.2 (5.6)	−1 (2.2)	1.1 (0.8)
	QT	147.1 (4.4)	−1.1 (1.7)	1.2 (1.1)
Time effect, *P*		**<.0001**	**<.0001**	.105
Group effect, *P*		.766	.704	.487
Interaction effect, *P*		.946	.200	.699

a Data are presented as mean (SD). Bold values indicate statistically significant *P* values. HT, hamstring; N/A, not applicable; QT, quadriceps tendon; ROM, range of motion.

**Table 4:** KT-1000 Knee Arthrometer Side-to-Side Difference Scores for the HT and QT Tendon Autograft Groups at 6, 12, and 24 Months After Surgery^*a*^

Variable	Measure	Group	6 Months (n = 104)	12 Months (n = 102)	24 Months (n = 97)
KT-1000 arthrometer, side-to-side difference, mm	Mean (SD)	HT	1.2 (0.9)	1.1 (0.9)	1.1 (0.8)
		QT	1.3 (1.4)	1.3 (1.2)	1.2 (1.1)
Normal, <3 mm	n (%)	HT	47 (92.2)	46 (93.9)	46 (93.9)
		QT	46 (86.8)	47 (88.7)	44 (91.7)
Nearly normal, 3-5 mm	n (%)	HT	4 (7.8)	3 (6.1)	3 (6.1)
		QT	7 (13.2)	6 (11.3)	4 (8.3)

a Data are presented as n (%). No knees were graded as abnormal (6-10 mm) or severely abnormal (>10 mm). HT, hamstring; QT, quadriceps.

**Table 5:** LSIs for Peak Isokinetic Knee Extensor (Quadriceps) and Flexor (Hamstring) Torque as Well as the 6 Single Hop Tests for the HT and QT Tendon Autograft Groups at 6, 12, and 24 Months After Surgery^*a*^

TimePoint	Group	Knee Extensor Torque LSI	Knee Flexor Torque LSI	SHD LSI	6 MTH LSI	THD LSI	TCHD LSI	LHD LSI	MHD LSI
6 months	HT	71.2 (16.8)	87.7 (12.1)	87.9 (10.2)	92 (8.9)	91.9 (5.9)	91.3 (7.2)	80.6 (13.8)	85.5 (14.4)
	QT	60.9 (14.8)	95.5 (12.8)	80.4 (16.3)	91.1 (11.1)	91.5 (8.7)	91.8 (9)	71.8 (17.4)	75.4 (17.6)
12 months	HT	85.3 (13.1)	93.8 (11)	93.4 (9.6)	93.7 (9.8)	94.9 (8.9)	94.4 (10.1)	91.9 (9.6)	93.7 (8.6)
	QT	77.3 (15.9)	102.9 (9.6)	91.2 (9.7)	94.3 (7.7)	94.9 (6.1)	93.9 (8.1)	86.6 (12.6)	89.9 (12.7)
24 months	HT	92.5 (8.2)	94 (8)	97.6 (3.5)	96.5 (5.8)	97.3 (4.7)	97.6 (5.8)	94.6 (9.6)	96.8 (7.1)
	QT	89.3 (10.9)	101.1 (5.1)	94.9 (8.4)	98.6 (5.2)	96.9 (5.6)	97.2 (6.1)	93.7 (8.9)	94.8 (8)
Time effect, *P*		**<.0001**	**<.0001**	**<.0001**	**<.0001**	**<.0001**	**<.0001**	**<.0001**	**<.0001**
Group effect, *P*		**.003**	**<.0001**	**.026**	.598	.678	.655	**.046**	**.007**
Interaction effect, *P*		**.001**	.469	.101	.502	.755	.321	**.027**	**.024**

a Data are presented as mean (SD). Bold values indicate statistically significant *P* values. HT, hamstring; LHD, single lateral hop for distance; LSI, limb symmetry index; MHD, single medial hop for distance; QT, quadriceps; SHD, single hop for distance; 6 MTH, 6-m timed hop; TCHD, triple crossover hop for distance; THD, triple hop for distance.

### Participation in Pivoting Sports

Of the 97 patients (HT = 49; QT = 48) available for the 24-month review, 85 patients (HT = 42; QT = 43) participated in pivoting sports before their ACL injury. Specifically, at 24 months, 32 (76%) patients in the HT group and 30 (70%) patients in the QT group were actively participating in their preinjury pivoting sports, with no significant difference in the rate of RTS (*P* = .505).

### Complications, Reoperations, and Reinjuries

Over the 24-month postoperative review period, 1 ipsilateral retear was observed (QT group at 22 months), while 2 contralateral tears were seen (QT group at 19 months; HT group at 23 months). Furthermore, several secondary surgical procedures were observed, including 5 in the HT group (1 manipulation under anesthesia for postoperative stiffness; 1 arthroscopic notch debridement for extension ROM issues; 2 knee arthroscopy for scar tissue; and 1 meniscal repair) and 6 in the QT group (1 arthroscopic notch debridement for extension ROM issues; 1 knee arthroscopy for scar tissue; 2 meniscal repairs; 1 tibial tubercle transfer; and 1 osteochondral autologous transplantation for a new cartilage injury to the lateral femoral condyle).

## Discussion

This study sought to comprehensively compare subjective, strength, and physical capacity measures, along with RTS, graft morbidity, and reinjury rates until 24 months after surgery in patients undergoing ACLR with an HT or QT autograft. Despite no apparent group differences in knee laxity, reinjury, or patient-perceived functional recovery over the period, a higher level of psychological readiness was reported in the HT group. Furthermore, graft-associated physical deficits were observed with greater hamstring strength LSIs in the QT cohort extending to 24 months, with greater quadriceps strength (and hop test) LSIs in the HT cohort extending to 12 months.

No group differences were observed in side-to-side laxity in the present study, and there was no further significant increase in side-to-side laxity after the initial 6-month laxity assessment, in support of the first hypothesis. Subsequently, whereas 76% of patients in the HT group and 70% of patients in the QT group who were participating in pivoting sports before their ACL injury had returned to these sports by 24 months, only 3 ACL reinjuries (1 ipsilateral rerupture and 2 contralateral ACL tears) had been observed up until 24 months. Although this may support the second hypothesis, it is acknowledged that this cohort requires ongoing review to better assess the longer-term failure rate of each graft construct. RTS outcomes in the present study were not dissimilar to those previously reported in a systematic review and meta-analysis published by Ardern et al^2^ who reported that only 65% of patients returned to their preinjury level of sports, with 55% returning to competitive sport. Most systematic reviews and meta-analyses comparing QT with HT and BPTB ACLR have reported no differences in laxity, reinjury, and/or revision surgery.^1,20,36,37,43,50,51^ However, other reviews have reported that QT ACLR resulted in less knee laxity^5,40^ and lower failure rates,^21,40^ which was not supported in the present study. Even though similar laxity and failure rates have also been reported in reviews that have compared HT and BPTB ACLR,^6,41^ reviews published by Li et al^32^ and Schuette et al^47^ reported that HT was inferior to BPTB ACLR in restoring knee joint stability, while Samuelsen et al^46^ reported that HT grafts failed at a higher rate than BPTB grafts. Of interest, a biomechanical cadaveric study published by Hart et al^16^ reported a comparative ultimate load to failure between HT, QT, and BPTB graft specimens, albeit the QT graft demonstrated more favorable structural properties compared with the other grafts and greater stiffness compared with the HT graft.

The present study demonstrated persistent isokinetic strength deficits that were related to the location of graft harvest—that is, residual quadriceps strength deficits that were greater in the QT group and persisted for 12 months, with residual hamstring strength deficits that were greater in the HT group and continued to 24 months—in support of the third hypothesis. A thorough assessment of the recovery of strength (and hop capacity) after QT ACLR compared with other graft constructs has been less reported. Reviews have reported comparable clinical and functional outcomes between QT ACLR and other grafts,^20,36,37,50^ although these are generally via PROMs and not objective assessment. A recent systematic review and meta-analysis comparing QT and HT ACLR published by Tan et al^51^ reported that HT ACLR was associated with better knee extensor strength outcomes. A systematic review and meta-analysis by Johnston et al^27^ investigated knee extensor and flexor strength outcomes after QT ACLR, comparing outcomes with the contralateral limb and patients undergoing alternative graft types. They reported that knee extensor strength LSIs after QT ACLR had not reached 90% by 24 months. However, knee extensor strength was weaker after QT ACLR when compared with HT ACLR. Although similar to BPTB at 5 to 8 months after surgery, knee flexor strength was greater than HT ACLR at 5 to 8 months. In RCTs comparing QT and HT ACLR, Martin-Alguacil et al^34^ reported better hamstring/quadriceps ratios after QT ACLR at 12 months, while Sinding et al^49^ reported that muscle strength was affected by autograft type, with QT ACLR demonstrating more pronounced knee extensor strength deficits. These findings are generally supported in the present study, albeit follow-up was undertaken 24 months after surgery.

Other comparative studies reporting strength outcomes have generally been nonrandomized and retrospective, with shorter postoperative follow-up periods. In a retrospective comparative study, Fischer et al^12^ reported significantly lower knee extensor strength and knee extensor LSIs and a higher hamstring/quadriceps ratio in the first 12 months after QT versus HT ACLR. The HT group demonstrated significantly lower hamstring strength LSIs. In another retrospective cohort study, Horteur et al^18^ reported large knee extensor strength side-to-side limb deficits at a mean 7 months after surgery, which were not different between QT and HT ACLR. In a cohort study, Lee et al^31^ reported better recovery of hamstring strength in patients undergoing QT versus HT ACLR. In a matched cohort study, Johnston et al^26^ reported significantly greater knee extensor strength deficits after QT versus HT ACLR at 6 months after surgery, albeit significantly greater knee flexor deficits were seen after HT ACLR. In another matched cohort study, Johnston et al^25^ reported that the significantly lower quadriceps strength and greater hamstring strength observed in QT versus HT ACLR 6 months after surgery persisted at 12 months.

The present study sought to perform a more comprehensive assessment of single-limb hop capacity after the strength assessment. Of interest, significant differences in hop test LSIs were observed up until 12 months in favor of the HT group. This did not support the fourth hypothesis, although it may have been related to the aforementioned group differences in quadriceps strength. In comparing QT and HT ACLR, a systematic review by Tan et al^51^ reported comparable SHD outcomes, while other studies have reported no differences in single-limb hop capacity between QT and either HT^39,44^ or BPTB^19^ ACLR. These outcomes are contrary to those of the present study. We acknowledge the limitations documented in employing LSIs to report strength and hop outcomes, such as the potential for LSIs to overestimate knee function^57^ and the presence of bilateral neuromuscular deficits reported in patients after unilateral ACLR.^8,13,22^ Nonetheless, previous research has highlighted the increased incidence of reinjury in patients not meeting 90% LSI criteria—particularly in peak isokinetic quadriceps strength and hop capacity—before RTS after ACLR.^14,29^ These outcomes may require consideration in graft selection and certainly provide a rationale for a more graft-specific rehabilitation intervention to ensure these deficits are appropriately addressed.

Similar to that reported in other systematic reviews and meta-analyses comparing QT ACLR with other graft types,^1,20,21,36,37,50,51^ no differences between groups were observed in the present study for commonly employed functional PROMs—such as the International Knee Documentation Committee, Lysholm, and Cincinnati Knee Rating System. Even though this was largely in support of the final hypothesis, of interest was the significantly better ACL-RSI score reported by the HT group up until and including 12 months after surgery. The association between greater psychological readiness after ACLR and knee laxity,^10^ as well as RTS,^10,11,17,30,59^ has been previously reported. A recent case-control study comparing QT and HT (semitendinosus and semitendinosus/gracilis) grafts reported no differences in ACL-RSI scores and kinesiophobia,^39^ but despite the better ACL-RSI scores observed in the HT group in the present study, there were no significant differences between groups in either laxity or the percentage of patients who were participating in preinjury pivoting sports. Webster et al^56^ reported that greater limb symmetry in the SHD positively affected psychological readiness for RTS. Whereas LSI differences for the SHD, LHD, and MHD were observed at 6 months (in favor of the HT group), LSI differences for the LHD and peak isokinetic quadriceps strength extended to 12 months, and this may also provide further rationale for the greater ACL-RSI scores reported by the HT group up until, and including, 12 months after surgery.

The present study demonstrated no group differences in donor-site morbidity, as reported primarily in the donor-site score and pain VAS at the graft site, albeit the donor-site score was only assessed from 6 months after surgery and may not reflect any burden in the earlier postoperative stages. Some published reviews comparing QT ACLR with other graft constructs have reported less donor-site morbidity when compared with BPTB ACLR,^36,43,50^ with Hurley et al^20^ reporting comparable donor-site morbidity. Although not as relevant to QT ACLR and not reported in the present study, other reviews comparing HT and BPTB ACLR have reported less risk of anterior knee pain and kneeling discomfort after HT ACLR.^6,32,41^ Anecdotally, even though lingering hamstring-related issues (soreness, cramping sensation, recurrent strains) can persist in those after HT ACLR and possibly be affected by the quality of rehabilitation, reported graft site morbidity was no worse than that associated with QT harvest in the present study.

Several study limitations should be acknowledged in the present study. First, although this RCT demonstrated a sound retention rate and a thorough subjective and objective patient assessment over the 24 months, it was not a double-masked study. Patients could not be blinded to their group allocation, while the varied location of scars between the 2 groups made it difficult to blind the physical therapist performing the assessments. Furthermore, patients were informed of their group randomization (surgery type) before surgery. However, despite only 1 patient's deciding to withdraw because of subsequently wanting the other graft type after randomization and being informed of that patient's group allocation, we acknowledge the limitation of making patients aware of their randomization before surgery. Second, the quality of rehabilitation can affect the recovery of strength and functional outcomes.^9^ Although a general rehabilitation plan was provided, this was a community-level cohort of patients who were provided guidance on rehabilitation and RTS from an array of physical therapists. Furthermore, there was no standardized preoperative rehabilitation period, which is now recommended as part of the standard management pathway for patients undergoing ACLR.^54^

## Conclusion

The 2 autograft groups compared well for PROMs (apart from the ACL-RSI), knee ROM, and laxity. However, greater hamstring strength LSIs were observed for the QT cohort, with greater quadriceps strength (and hop test) LSIs in the HT cohort. Longer-term review will continue to evaluate RTS and later-stage reinjury between the 2 graft constructs.
